# Fast and adaptive dynamics-on-graphs to dynamics-of-graphs translation

**DOI:** 10.3389/fdata.2023.1274135

**Published:** 2023-11-17

**Authors:** Lei Zhang, Zhiqian Chen, Chang-Tien Lu, Liang Zhao

**Affiliations:** ^1^Department of Computer Science, Virginia Tech, Falls Church, VA, United States; ^2^Department of Computer Science and Engineering, Mississippi State University, Mississippi, MS, United States; ^3^Department of Computer Science, Emory University, Atlanta, GA, United States

**Keywords:** graph, ESN, reservoir computing, GNN, NAS

## Abstract

Numerous networks in the real world change with time, producing dynamic graphs such as human mobility networks and brain networks. Typically, the “dynamics **on** graphs” (e.g., changing node attribute values) are visible, and they may be connected to and suggestive of the “dynamics **of** graphs” (e.g., evolution of the graph topology). Due to two fundamental obstacles, modeling and mapping between them have not been thoroughly explored: (1) the difficulty of developing a highly adaptable model without solid hypotheses and (2) the ineffectiveness and slowness of processing data with varying granularity. To solve these issues, we offer a novel scalable deep echo-state graph dynamics encoder for networks with significant temporal duration and dimensions. A novel neural architecture search (NAS) technique is then proposed and tailored for the deep echo-state encoder to ensure strong learnability. Extensive experiments on synthetic and actual application data illustrate the proposed method's exceptional effectiveness and efficiency.

## 1 Introduction

Graphs are commonly used as universal representations of real-world things, including social networks, brain functional connections, and molecular topology. Real-world networks generally exhibit patterns in their dynamics, which may be classed as “dynamics **on** graphs” and “dynamics **of** graphs.” The former stresses the time-evolving patterns of the entities' activity, which can be proven explicitly through the observable node attributes, whereas the latter emphasizes the underlying change in the topological structure of the network. Both forms of dynamics appear in real-world graphs, and it is tremendously advantageous to understand their linkage and transformation. In social networks, for instance, it is crucial to investigate how the node-level behaviors might affect time-evolving connectivities (Gao et al., 2022). In neuroscience, it is essential to examine how the co-activation of many neurons increases their physical nerve connections (Ma et al., 2019). In recent years, a substantial amount of work and knowledge has been devoted to “dynamics graphs,” a mix of “dynamics on graphs,” and “dynamics of graphs.” Dynamic graph embedding methods, for instance, compute dynamic node embedding by aggregating messages from nodes' neighborhoods, which requires the input of both node signals (i.e., dynamics on graphs) and graph topology (i.e., dynamics of graphs) (Taheri et al., 2019; Pareja et al., 2020; Sankar et al., 2020). In practice, however, it is typically quite difficult to directly measure all the edges to immediately perceive the entire graph. Instead, it is considerably more frequent and economical to deduce the underlying network structure from the node signals. Therefore, we propose the task that transfers “dynamics on graphs” to “dynamics of graphs” (in short, “**on-to-of**” task) to map the two individual spaces. Existing on-to-of efforts can be divided into two distinct categories. The first class of approaches discretizes continuous node signals before implementing message passing on a fully connected (Pareja et al., 2020; Yang et al., 2021), resulting in a severe loss of information. The second category encodes full time series directly into their embeddings and then calculates the correlation between these embeddings using standard metrics such as cosine similarity (Hlinka et al., 2013; Tupikina et al., 2016; Kipf et al., 2018; Graber and Schwing, 2020). However, such metrics usually imply strong priors and thus cannot adapt to more complicated on-to-of mappings.

This study focuses on the on-to-of task, which cannot be effectively addressed by existing solutions due to the following challenges: **(1) Difficulty in jointly extracting features from node dynamics while learning the dynamic relationships in a graph**. The challenge necessitates that transformation patterns be considered in both time and graph dimensions. Moreover, these two dimensions are not independent, necessitating the need for a framework that can facilitate the combined evolution of node and edge dynamics. **(2) Absence of an effective and scalable framework for graph dynamics encoding over a continuous long time duration with a high sampling rate**. The inference of dynamic graph topology necessitates fine-grained, long-term knowledge on graph dynamics. Existing efforts for dynamic network embedding and representation learning are unable to efficiently manage extended time series of node attribute data. **(3) Dilemma between learnability and efficiency of models**. Modeling the complex mapping between node and edge dynamics demands models with a high capacity for learning. Compared to optimizing a model fitted to specific data, optimizing a highly flexible, highly learnable model is typically time-consuming.

To address the above challenges, we present a novel framework based on echo-state network (ESN) and neural architecture search (NAS). Specifically, a deep echo-state network is proposed to efficiently encode the continuous time series of node attributes into dynamic edge embeddings. The architecture of the ESN is automatically tuned by NAS in a self-supervised manner. The application of the ESN makes the framework extremely efficient and scalable when dealing with continuous node signals. The NAS module enables the framework to be adaptive to varying data with minimum priors and ensures good results. The contributions of this study are as follows: **(1) Propose the first NAS method for ESN**. To efficiently encode the continuous node signals, we propose to use ESN as the the encoder and tune its architecture with NAS methods. To the best of our knowledge, this is the first work that defines the search space of ESN and optimizes its architectures automatically for downstream tasks. **(2) Design a novel generic framework for mapping between “dynamics on graphs” and “dynamics of graphs”**. Different from existing studies, the proposed framework is generic and does not depend on specific mapping priors. We show that ESN and NAS complement each other and fit the problem well. First, ESN is efficient and scalable but suffers from low performance. Second, NAS makes the model adaptive to target data but is expensive to train for large regular. The combination of NAS and ESN yields a good balance between scalability and performance and is well suited for the generic “dynamics on graphs” and “dynamics of graphs” translation task. **(3) Conduct extensive experiments for performance and efficiency evaluations**. The proposed method was evaluated on both synthetic and real-world application data. The results demonstrate that the proposed approach runs significantly more efficiently and exhibits better performance than the baseline methods.

## 2 Related work

### 2.1 “Dynamics on graphs” and “dynamics of graphs”

The studies on graph structure learning (GSL) and dynamic graph embedding studies are the most relevant to “dynamics on graphs” and “dynamics of graphs.” The graph topology learning strategies in GSL can be classified into metric-based, neural approaches, and direct approaches (Zhu et al., 2021). Most existing studies are metric-based (Du et al., 2012; Hlinka et al., 2013; Graber and Schwing, 2020) which rely on strong priors of the graph definition. The direct approaches are not related to the on-to-of task because the optimization for an extra downstream task is needed. The neural approaches can be used for the generic on-to-of task. Only recently, several neural approaches have been proposed for dynamic graph data and dynamic graph topology (Graber and Schwing, 2020; Rossi et al., 2020). These research have a greater emphasis on strengthening the graph neural network module and solely use conventional 2D-CNNs or RNNs to encode continuous graph signals. Furthermore, the GSL modules highly rely on *ad-hoc* heuristic models that are designed under strong priors. For example, Tupikina et al. (2016) assumed that the graph is a temporal correlation matrix; Graber and Schwing (2020) tried to recover the underlying physical interaction relationship with temporal dependencies; Hlinka et al. (2013) focused on inferring entropy graphs; Kipf et al. (2018) assumed a static graph is the cause of the dynamics; Du et al. (2012) tried to recover the maximum likelihood diffusion graph of cascades of events. Unlike previous research, our proposed framework is designed to be highly adaptive and does not rely on pre-processing or the usually unknown on-to-of mapping mechanism.

### 2.2 Echo-state networks

Reservoir computing is a computational paradigm suited for temporal/sequential data processing (Verstraeten et al., 2007; Lukoševičius and Jaeger, 2009). Though different implementations of reservoir computing exist in studies (Tino and Dorffner, 2001; Maass et al., 2002), the echo state network (ESN) is the most widely known model, with a strong theoretical ground (e.g., Gallicchio and Micheli, 2011; Manjunath and Jaeger, 2013; Massar and Massar, 2013; Tiňo, 2018) and plenty of successful applications reported in studies (Bacciu et al., 2014; Crisostomi et al., 2015; Palumbo et al., 2016). In recent years, ESNs have been applied to static graph data (Gallicchio and Micheli, 2020) and even with extensions to dynamic graphs where only the node labels change over time (Tortorella and Micheli, 2021). ESNs have not been used for the more complicated on-to-of task where two types of dynamics exist. While it is well known that regular neural networks' architecture plays a vital role in their performance, the effect of the ESNs' architecture still remains unclear. The pioneering studies of deep ESN has been discussed in Gallicchio and Micheli (2017). While deep ESNs have shown potential on efficiently processing temporal data, the initialization of deep ESNs is still underexplored (Jaeger, 2002). Pre-training schemes such as PSO were used to alter the ESN topology manually in a trial-and-error manner (Chouikhi et al., 2017). Our study is different from this line of research, as we first propose to initialize the echo-state network automatically with neural architecture search.

## 3 Problem definitions

In this section, basic concepts and problem definitions are introduced.

Definition 3.1 (Dynamics **on** graphs). In a graph with *V* nodes, the dynamics on graph are defined as the multivariate time series sensed continuously on all the nodes denoted as *S* = {*S*^(1)^, *S*^(2)^, ⋯ , *S*^(*V*)^}, where *S*^(*i*)^ is the node signal for node *i*.

Definition 3.2 (Dynamics **of** graphs). For a graph with a maximum number of *V* nodes, the dynamics of graphs is an ordered sequence of separate weighted graphs $A=\left\{A_{1},A_{2},\cdots \;,A_{m}\right\}$, where $A_{k}\in {\mathbb{R}}^{V\times V}$ corresponds to an incidence matrix weighted or adjacency matrix.

In reality, it is usually very difficult to directly measure all the edges in order to sense the whole graph directly. Instead, it is much more efficient and common to sense the node signals on the nodes to infer the underlying graph structure. We propose the problem which maps the “dynamics on graphs” to “dynamics of graphs” (in short, the *on-to-of* problem) for mapping the two spaces.

Definition 3.3 (The **on-to-of** task). We assume that the dynamics on graphs data *S* and dynamics of graph A are all evenly segmented: *S* = {*S*_1_, *S*_2_, ⋯ , *S*_*m*_}, $A=\left\{A_{1},A_{2},\cdots \;,A_{m}\right\}$, where the $A_{k}$ is the ground truth underlying dynamics of graphs in the *k*−th segment of dynamics on graphs, i.e., *S*_*k*_. The on-to-of task is to infer a function to map between S and A: F:S→A.

For convenience, we denote *S* = {*S*_1_, *S*_2_, ⋯ , *S*_*m*_} for *m* time series, and *S* = {*S*^(1)^, *S*^(2)^, ⋯ , *S*^(*V*)^} for *V* nodes. $S_{k}^{\left(i\right)}$ is the *i*−th node's *k*−th time series segment. $S_{k}^{\left(i\right)}$ can be further defined as discrete time series with length *l*: $S_{k}^{\left(i\right)}=\left\{s_{k,1}^{\left(i\right)},s_{k,2}^{\left(i\right)},...,s_{k,l}^{\left(i\right)}\right\}$.

It is important to note that the on-to-of task is different from most of the dynamic graph studies because only the node signals are used as input, and the graph structure is the output. In most dynamic graph studies, the graph structure must be used as well in a graph neural network (Pareja et al., 2020; Yang et al., 2021). The on-to-of task is also different from the more commonly studied GSL problem (Zhu et al., 2021) where the evolving graph topology (i.e., dynamics of graphs) is optimized toward optimizing other downstream tasks (Graber and Schwing, 2020). In the on-to-of task, though, recovering the dynamic graph topology is the task itself.

## 4 Methodology

**Figure 1:**
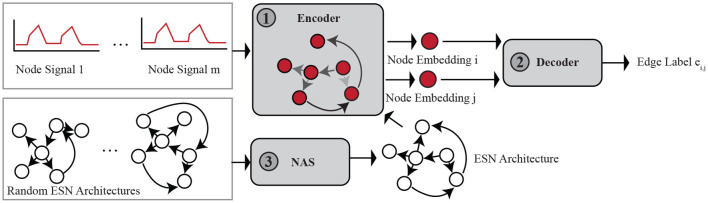
Adaptive deep echo-state framework for the on-to-of task.

To solve the on-to-of task and address the challenges, we propose a new adaptive deep echo-state framework for graph dynamics transformation in this section. The adaptive deep echo-state framework (AD-ESN) mainly includes three modules as demonstrated in Figure 1. To solve the challenge of lacking scalability, we extend echo state network (ESNs) and propose a deep ESN-based graph dynamics encoder (module ① in Figure 1). In order to improve the performance and make the model adaptive, we propose a novel idea of using NAS on ESNs (module ③ in Figure 1). This solution not only solves the challenge of lacking model assumptions for the on-to-of task but also remedies the shortage of vanilla ESNs that the performance is poor. ESN and NAS together enable us to learn meaningful representations of arbitrary node signals in a graph. We used an attention-based dynamic graph topology decoder (module ② in Figure 1) for mapping the node embeddings to edge labels as detailed in Section 4.2. While NAS is a powerful technique, it can also be extremely slow, which does not match our original intention of proposing an efficient on-to-of task solver. We solved this issue by proposing a surrogate loss and using a gradient-based optimization algorithm which is much faster to solve.

### 4.1 Efficient continuous time node signal encoding

We propose an adaptive deep ESN-based encoder for learning the representation of the dynamics of graphs. The input data are non-linearly embedded into a higher dimensional feature space, where the original problem is more likely to be solved linearly according to Cover's Theorem (Cover, 1965). With proper settings, the dependencies of our ESN-based graph dynamics encoder on the initial conditions are progressively lost, and the state of the network asymptotically depends only on the driving input signal.

**Figure 2:**
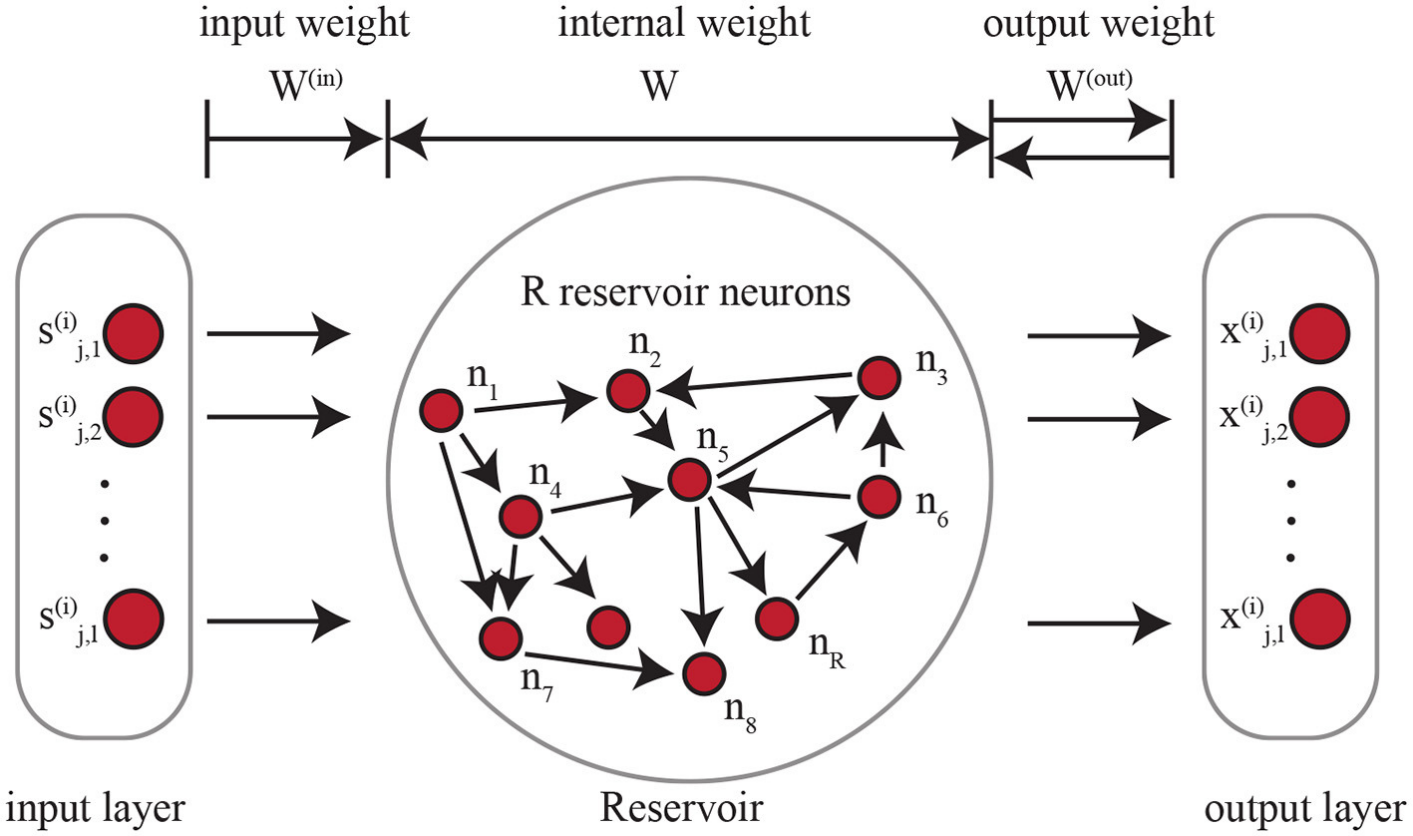
Echo state network. The architecture of the reservoir is optimized with NAS.

The ESN-based encoder can be considered as a RNN where all of the weights are randomized and untrained. As shown in Figure 2, there are input weight, internal weight, and output weight. On the left side of the figure, each timestamp in the input time series is considered as an input node. *W*^(*in*)^∈ℝ^*d*×*R*^ is the weight that maps each input node with *d*-dimensional signal to the internal reservoir neurons. Every time a new timestamp $S_{k}^{\left(i\right)}$ is fed in, the reservoir neurons are affected by not only the input data but also the current state of all the neurons in the ESN. Similarly, the output weight *W*^(*out*)^ connects the state of the reservoir neurons to the outputs. The dynamics of the ESN can be represented as $r_{t}=\sigma \left(Wr_{t-1}+W^{\left(in\right)}s_{k,t}^{\left(i\right)}\right)$, where σ is a non-linear activation function, *r*_*t*_ is the current state of the ESN reservoir at time *t*, *W*∈ℝ^*R*×*R*^ is the weight among the *R* reservoir neurons (shown in Figure 2). The architecture of the ESN-based encoder is tuned by NAS as detailed in Section 4.3 and Section 4.4.

For a time series segment with length *l*, we denote R as the ESN's reservoir, and $x_{k}^{\left(i\right)}$ as the representation of the *k*-th time series segment of node *i*. $x_{k}^{\left(i\right)}$ can be calculated as the final hidden representation in the ESN that can be computed recursively as shown in Eq. (1):


(1) $$\begin{array}{l} x_{k}^{\left(i\right)}=W^{\left(out\right)}ℛ(S_{k}^{\left(i\right)})=W^{\left(out\right)}\sigma ((Wr_{k,l-1}^{\left(i\right)}+W^{\left(in\right)}s_{k,l}^{\left(i\right)}))\\ \;=W^{\left(out\right)}\sigma (W\sigma (Wr_{k,l-2}^{\left(i\right)}+W^{\left(in\right)}s_{k,l-1}^{\left(i\right)})+W^{\left(in\right)}s_{k,l}^{\left(i\right)}). \end{array}$$

One of the most essential features in ESN that we utilize is that the input weight *W*^(*in*)^ and internal weight *W* are *randomized* and will not be optimized during the training process. Only the output weight *W*^(*out*)^ will be trained on the labeled data. The feature that *W*^(*in*)^ and *W* are not trained makes ESNs efficient and scalable. At the same time, ESNs suffer from poor performance compared with similar modern recurrent neural networks that are optimized with backpropagation through time (BPTT).

In conclusion, the ESN does not *learn* the representation of the input time series. It is directly applied to the sequential input data and maps the input into a high-dimensional space. While ESN is efficient, tuning its initial state is highly dependant on domain experts' experiences. It is highly desired if ESNs can be automatically tuned before its trained on the labeled data.

### 4.2 Dynamic graph topology decoding

The proposed attention-based dynamic graph topology decoder aims to infer time-evolving edge features or connectivities, ensuring that the on-to-of task inference is scalable and general without bias. Within the *k*-th time series segment, the dynamics on graphs now are represented as $X_{k}=\left\{x_{k}^{\left(1\right)},x_{k}^{\left(2\right)},\cdots \;,x_{k}^{\left(V\right)}\right\}$ on *V* nodes. We define the attention coefficients between node *i* and node *j* as the edge label (dynamics of graphs) we try to infer: $e_{k,i,j}=a\left(x_{k}^{\left(i\right)},x_{k}^{\left(j\right)}\right)$, where *a* is the attention mechanism with shared weights. As there is already a shared learnable linear transformation layer *W*_*out*_ in the ESN before getting the embeddings, an external linear transformation is omitted before using the attention mechanism. In practice, the attention *a* is implemented as a single head GAT (Veličković et al., 2018) (denoted as function *g*) parameterized with *W*^(*G*)^ defined on a fully connected graph. Now, we can express the on-to-of task as $A_{k,i,j}=\underset{\left(W^{\left(out\right)},W^{\left(G\right)}\right)}{F}\left(S_{k}^{\left(i\right)},S_{k}^{\left(j\right)}\right)=\frac{exp\left(\sigma \left(g\left(W^{\left(out\right)}R\left(S_{k}^{\left(i\right)}\right)||W^{\left(out\right)}R\left(S_{k}^{\left(j\right)}\right)\right)\right)\right)}{\underset{n\in V}{\sum }exp\left(\sigma \left(g\left(W^{\left(out\right)}R\left(S_{k}^{\left(i\right)}\right)||W^{\left(out\right)}R\left(S_{k}^{\left(n\right)}\right)\right)\right)\right)}$.

### 4.3 The neural architectures of echo-state networks

Given a class of neural networks, the first step of NAS is to define the *search space*. Take CNN models as an example. The size and number of the convolution kernels, the number of clusters, and the choice of activation functions in most CNN models are all hand-crafted. NAS is the process that optimizes the neural network architecture automatically such that it performs best on the data after training. On ESNs, it is easy to see that by carefully assigning the connectivity represented as the weights in *W*^(*in*)^ and *W*, regardless of the weights, the ESN can become deep-layered architectures (Gallicchio et al., 2018).

As no NAS studies have been proposed for ESNs, we propose ESN architecture search, the first attempt of using NAS on ESNs for graph dynamics learning. The goal is to automatically learn the ESN architecture so that it will achieve a good performance in downstream tasks.

The following is the search space that we define for ESNs.

Definition 4.1 (The search space of echo-state networks). The search space of echo-state networks is defined as follows:

- The connectivity from the input to ESN's reservoir neurons *A*^(*in*)^∈ℝ^*d*×*R*^ where $A_{i,j}^{\left(in\right)}=\left\{0,1\right\}$. $A_{i,j}^{\left(in\right)}=\left[W_{i,j}^{\left(in\right)}\neq 0\right]$ ^1^
- The connectivity between ESN's reservoir neurons *A*∈ℝ^*R*×*R*^ where *A*_*i, j*_ = {0, 1}. *A*_*i, j*_ = [*W*_*i, j*_≠0].

For simplicity, we denote *A*^(*R*)^ = [*A*^(*in*)^, *A*] as the ESN's architecture. The choice of activation functions and the number of reservoir nodes are also hyperparameters in the search space but can be set according to general rules (will be discussed in Section 5.2.

### 4.4 Deep-echo-state architecture optimization

A rigorous optimization loss function for optimizing AD-ESN can be expressed as follows:


(2) $$\begin{array}{l} A^{\left(R\right)}=\underset{A}{\arg\min}\;ℒ(W^{(out)*},W^{(G)*},A),\\ ℒ(W^{\left(out\right)},W^{\left(G\right)},A)=\sum_{0<k<m}\sum_{i\neq j}|F_{\left(W^{\left(out\right)},W^{\left(G\right)},A\right)}(S_{k}^{\left(i\right)},S_{k}^{\left(j\right)})-A_{k,i,j}|,\\ \text{where }[W^{(out)*},W^{(G)*}]=\underset{{}_{\left[W^{\left(out\right)},W^{\left(G\right)}\right]}}{\arg\min}ℒ(W^{\left(out\right)},W^{\left(G\right)},A), \end{array}$$

here, $F_{\left(W^{\left(out\right)},W^{\left(G\right)},A\right)}$ represents the graph topology decoder with a fixed ESN architecture *A*.

However, the loss in Eq. (2) is extremely expensive to solve as it requires doing the bi-level optimization on the original on-to-of task. One common practice in NAS studies is to propose a surrogate loss that is much easier to solve. Inspired by self-supervised learning, instead of optimizing the ESN for the empirical loss in Eq. (2), we decouple the ESN-based encoder and the graph topology decoder, then define a surrogate loss function for the prediction performance of the ESN-based encoder. Given the time series data $S^{\prime }=\left\{s_{1}^{\prime },s_{2}^{\prime },\cdots \;,s_{m}^{\prime }\right\}$ sampled from the whole data *S*, the NAS problem with the surrogate loss is defined in Eq. (3).


(3) $$\begin{array}{l} A^{\left(R\right)}=\underset{A}{\arg\min}{ℒ}_{s}(W^{\left(pred*\right)},A),\\ {ℒ}_{s}(W,A)=\sum_{0<t<m-1}|{{ℛ}^{\prime }}_{W,A}({S^{\prime }}_{0:t})-{s^{\prime }}_{t+1})|,W^{\left(pred*\right)}=\\ \underset{W}{\arg\min}{ℒ}_{s}(W,A^{\left(R\right)}), \end{array}$$

where the previous *W*^(*out*)^ is replaced with *W*^(*pred*)^. Differing from *W*^(*out*)^, *W*^(*pred*)^ transforms the internal states of the ESN reservoir into a representation that shares the same dimensionality as the time series data. $R_{W,A}^{\prime }$ denotes the surrogate reservoir with *W* as the output weight and *A* as the architecture. Unlike R, $R^{\prime }$ functions as a reservoir that acts as a self-regression function. The rationale behind this surrogate loss aligns with NRI (Kipf et al., 2018): effective architectures enable accurate forecasting. To efficiently address the problem in Eq. (3), we employ the reparameterization trick and sample ESN graph connections using Gumbel-Softmax. For each data batch, the ESN architecture is sampled based on the continuous *A*^(*R*)^. The optimization of the ESN's prediction weight *W*^(*pred*)^ is achieved through regular backpropagation. The optimization of the ESN's architecture parameter *A*^(*R*)^ follows a simple heuristic, optimizing the validation loss by assuming that the current *W*^(*pred*)^ is identical to *W*^(*pred*′^). Once the optimization is completed, to establish the discrete topology in the ESN, we retain the edge labels (presence or absence) by applying a threshold λ. Additional details regarding the NAS process can be found in the Supplementary material. The outlined procedures are described in Algorithm 1.

**Algorithm 1:** Bi-level optimization for the encoder's architecture.

1: initialize *A*^(*R*)^ in continuous space
2: **while** Early stopping criterion is not met **do**
3: **for** *e* in epoch **do**
4: **for** minibatch in training and validation data **do**
5: //Sample discrete ESN according to *A*^(*R*)^
6: *A*^(*R*)^~*Gumbel*(0, 1)
7: Initialize *W*^(*in*)^ and *W* according to *A*^(*R*)^
8: // Update the ESN's predict weights on the training set
9: $W=W-\eta_{W}\nabla_{W}L_{tr}\left(W,A^{\left(R\right)}\right)$
10: // Update the ESN's topology parameters *A*^(*R*)^ on the validation set
11: $A^{\left(R\right)}=A^{\left(R\right)}-\eta_{A^{\left(R\right)}}\nabla_{A^{\left(R\right)}}L_{val}\left(W,A^{\left(R\right)}\right)$
12: **end for**
13: **end for**
14: **end while**

The time complexity of our ESN-based encoder module is O(Rl), where *R* is the number of internal reservoir neurons and *l* is the length of the time series. Before our method, the standard way of handling time series data with recurrent neural networks is BPTT (backpropagation through time), which is $O\left(R^{2}l\right)$ (Williams and Zipser, 1995). The time complexity of the graph topology decoder can be expressed as $O\left(V^{2}\right)$. This is attributed to the pairwise computation of node hidden feature vectors which is inevitable. There is also a NAS module in our framework that takes time, while a vanilla RNN model does not require it. However, the NAS is meant to automate the fine-turning process that was originally performed by humans, which normally takes a longer time. Furthermore, our proposed NAS process is independent of the supervised-learning process and only runs on small sampled unlabelled data.

## 5 Experiments

### 5.1 Datasets

The proposed AD-ESN framework and baseline methods are tested on five datasets including two synthetic and three real-world datasets: **Syn-Coupled** (Kipf et al., 2018) is a physical simulated dataset for phase-coupled oscillators. Each node is an oscillator that is coupled with its neighbors according to a dynamic graph. **Syn-Chaotic** is another physical simulated dataset. Each node is defined as a chaotic time series. The ground truth dynamic graphs are defined as real-time correlation graphs. **Brain** is a real-world brain fMRI data. The dynamic graphs represent the functional connectivity between brain regions. The node signals represent the BOLD (blood oxygenation level dependent) time series in each of the brain regions. **Social** (Gao et al., 2019) is a real-world social media dataset. The node signals are forum users' activities. The dynamic graphs are users' accumulated transition graphs. **Protein** (Anand and Huang, 2018) is a real-world protein folding data. The node signals contain the amino acids' 3-dimensional dynamic coordinates. The dynamic graphs are the protein's connectivity during the folding process. For Syn-Chaotic, Forum, and Brain datasets, the edges have dynamic weights, such that the “dynamics of graphs” are represented as affinity matrices. We evaluate the results with average MAE and RMSE. For Syn-Coupled and Protein datasets, the “dynamics of graphs” are represented as adjacency matrices. The results are evaluated with accuracy (Acc) following (Kipf et al., 2018). We also report the recall (sensitivity) rate as it is important for the model to have fewer false negatives, i.e., discover the edge if it exists. More detailed data descriptions can be found in Supplementary material.

### 5.2 Experiment settings

All the models are trained with the ADAM optimization algorithm. For each of the datasets, 80% of the data are used as the training set, 10% for testing, and 10% for validation. The architecture of the ESN is optimized on 10% of the training data with a gradient-based NAS algorithm. For an input of size *d*, to remember τ time points in the past, the number of ESN nodes is set as *d*×τ (Lukoševičius, 2012). The randomly generated ESN weights are normalized to meet a standard called echo state property (ESP). More implementation details can be found in Supplementary material.

To show AD-ESN's strengths in terms of adaptability and scalability, we compare it with two baselines: *LSTM-Att* utilizes LSTM as encoder and GAT as decoder. *ESN* adopts vanilla ESN as encoder and GAT as decoder. Additionally, we compare AD-ESN with two recent SOTA approaches for graph inference: *NRI* (Kipf et al., 2018) uncovers the relation graph by learning a variational auto-encoder (VAE). *dNRI* (Graber and Schwing, 2020) is similar with NRI but encodes the temporal dependence with LSTM. At last, two simple comparison methods are also used: *Pre-step* simply determines the relations between nodes as the previous status in the last segment. *Siamese* (Mueller and Thyagarajan, 2016) encodes the time series with LSTM and decode the graph dynamics with a siamese feed-forward neural network.

### 5.3 Performance and adaptability analysis

**Figure 3:**
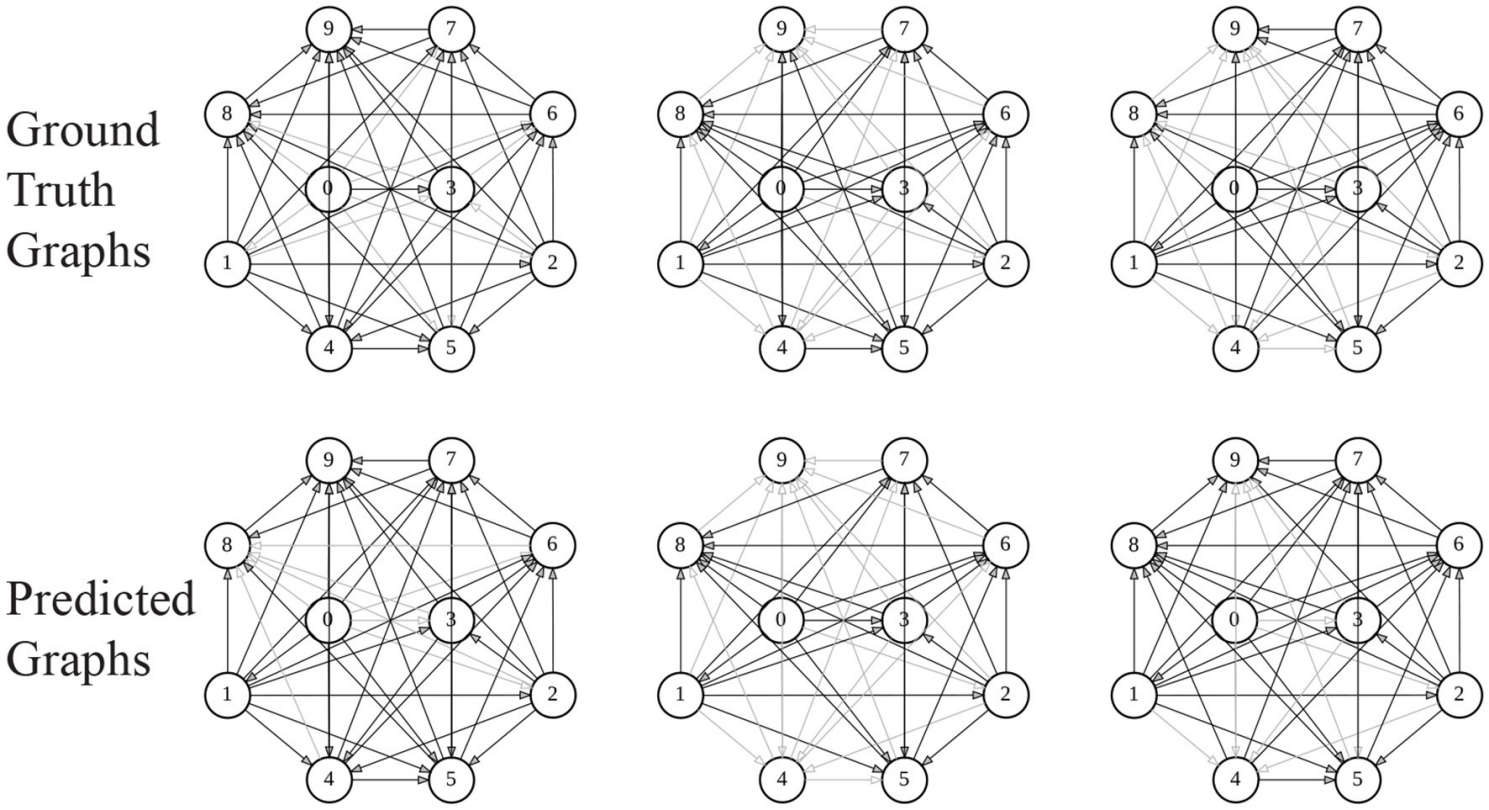
Visualization of graphs. Darkness of the edges reflects their weights.

Table 1 summarizes the results of all the models on all the datasets. We observed that AD-ESN achieves overall the best performance on all five datasets with different data scales and underlying priors, which indicates the exceptional adaptability of AD-ESN over the baselines. Specifically, AD-ESN surpasses ESN in all tasks, highlighting the efficacy of neural architecture searches. Some of the baseline models can perform well on a restricted set of tasks but fall short on others, which means they are much more sensitive to datasets. For simulated coupled oscillator data, we first tested all the methods on a sampled dataset with the same initialization circumstances as NRI (Syn-Coupled-1) (Kipf et al., 2018), then another dataset with a different configuration (Syn-Coupled-2). It can be seen that NRI and dNRI perform better than AD-ESN on Syn-Coupled-1 but fails miserably on Syn-Coupled-2 when the hyperparameters are not fine-tuned. Our proposed AD-ESN architecture, on the other hand, performs slightly worse than NRI and dNRI on Syn-Coupled-1 but significantly better than all the rest comparison approaches. This serves as a noteworthy illustration showcasing both the capabilities and limitations of our proposed AD-ESN framework. In the case of a dataset such as Syn-Coupled-1, for which existing methods (e.g., NRI) have been specifically designed and optimized, AD-ESN may not necessarily surpass these established methods. Nevertheless, its notable strength lies in its adaptability across a diverse array of datasets, consistently delivering satisfactory performance even when other methods prove ineffective. On the remaining datasets, our proposed AD-ESN consistently outperforms the other methods. Only two of the ESN-based algorithms are scalable to be trained on the Brain dataset since the time-series of nodes are excessively long. The predicted graphs using AD-ESN are compared to the ground truth graphs from the Syn-Chaotic dataset in Figure 3. The adaptability of AD-ESN is enabled by the proposed deep-echo-state graph dynamics encoder which is automatically altered with NAS in a self-supervised manner.

**Table 1:** Performance comparison.

	**Dynamic edge binary classification**						**Dynamic edge weight estimation**					
**Datasets**	**Syn-Coupled-1**		**Syn-Coupled-2**		**Protein**		**Syn-Chaotic**		**Forum**		**Brain**	
**Metrics**	**Acc**	**Recall**	**Acc**	**Recall**	**Acc**	**Recall**	**RMSE**	**MAE**	**RMSE**	**MAE**	**RMSE**	**MAE**
Pre-step	51.2	72.5	47.7	70.3	64.5	68.7	0.036	0.033	0.33	0.067	0.8	0.505
LSTM-Att	51.4	62.5	51.7	49.9	53.3	45.3	0.036	0.033	0.26	0.047	-	-
Siamese	66.2	14.6	53.2	11.3	50.0	47.3	0.039	0.035	0.26	0.052	-	-
ESN	50.0	48.6	50.4	48.3	50.0	42.4	0.031	0.03	0.49	0.091	0.93	0.562
NRI	**94.6**	**91.5**	49.9	33.5	49.9	48.2	**0.029**	**0.026**	0.43	0.075	-	-
dNRI	94.4	91.4	56	33.7	56.3	62.6	**0.029**	**0.026**	0.49	0.088	-	-
AD-ESN	92.2	90.9	**74.4**	**83.6**	**67.8**	**71.4**	**0.029**	**0.026**	**0.26**	**0.05**	**0.67**	**0.442**

“-” denotes that the model is not trainable on our hardware. The best performance is indicated by the use of bold numbers.

### 5.4 Scalability analysis

**Figure 4:**
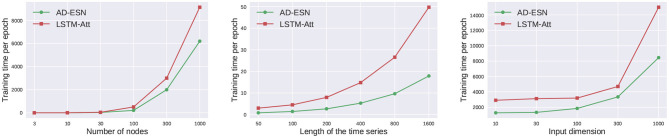
Scalibility Analysis. The ESN encoder makes the framework much more efficient and scalable.

Table 2 shows a comparison of LSTM-Att and AD-ESN models' GPU RAM usage on synthetic data. The hidden dimensions of both models are the same. The LSTM-Att model utilizes more RAM than the AD-ESN model when used to process long time series. The advantage increases when the time series data become longer because RNN-based models (e.g., LSTM, GRU) require memory to store the gradients for each timestamp backpropagation, whereas ESNs do not. A training time comparison between models employing the ESN-based encoder and its LSTM-based counterpart (LSTM-Att) is shown in Figure 4. The number of parameters in the two models is fixed to be the same to make a fair comparison. The training cost of the NAS process is negligible compared with the actual training time due to the efficient gradient-based bi-level optimization and the surrogate loss. While the ESN-based encoder is only one component of the whole framework, the training time of the whole AD-ESN framework is much shorter in all scenarios, especially when the length of time series increases, which coincides with the time complexity analysis in Section 4.4.

**Table 2:** GPU usage test results.

**Model**	**L**	**D**	**Node #**	**BS**	**GPU memory**
LSTM-Att	500	100	50	128	8,893 MB
AD-ESN	500	100	50	128	6,425 MB
LSTM-Att	1000	10	50	128	6,581 MB
AD-ESN	1000	10	50	128	1,647 MB
LSTM-Att	5000	1	50	128	N/A
LSTM-Att	5000	1	50	↓ 32	7464 MB
AD-ESN	5000	1	50	128	**2227 MB**

L, Time Series Length; D, Input dimension; BS, Batch size. N/A means the GPU memory is not enough for this setting. The best performance is indicated by the use of bold numbers.

## 6 Conclusion

This research has focused on solving the generic “dynamics on graphs” to the “dynamics of graphs” translation tasks without knowing what type of mapping was employed. To do so, we have proposed a generic ESN-based framework with NAS that can automatically tune its architecture based on the input continuous node signal data in a self-supervised manner. To the best of knowledge, this is the first study that combines ESN and NAS. This combination enables the framework to achieve a compelling trade-off between the efficiency and neural architecture flexibility. Experiment results attest that our AD-ESN framework can successfully uncover the underlying on-to-of mappings on different types of data. The employment of ESN and NAS has been proven to be surprisingly effective and makes the framework highly versatile and scalable.

## Data availability statement

The original contributions presented in the study are included in the article/Supplementary material, further inquiries can be directed to the corresponding author.

## Author contributions

LZhao, and ZC conceived of the presented idea. LZhan designed the model and conducted the experiments. LZhao provided datasets and problem definition, and contributed the domain knowledge. ZC and C-TL offered the feedback on the proposed method and writing. All authors contributed to the article and approved the submitted version.

## Footnotes

^1^Please note that *A* represents the graph structure of ESNs, while A represents the graph structure of the target graphs (dynamics of graphs).
