# Supplementary material for: Fast and adaptive dynamics-on-graphs to dynamics-of-graphs translation
Source: Front Big Data. 2023 Nov 17;6:1274135. doi: 10.3389/fdata.2023.1274135 (PMC10691542; doi:10.3389/fdata.2023.1274135)
Supplement: Supplementary file 1 [file Presentation_1.zip › frontier_ESN_Supplementary.pdf]

Supplementary Material

1 A. DATASETS

1.1 Syn-Chaotic

This synthetic dataset is for verifying if the proposed model can learn a pre-defined mapping function without any prior knowledge. We assume each node generates a chaotic time series that exhibits complex nonlinear behavior but with a deterministic property. The synthetic chaos time series can be generated from the Mackey-Glass function Mackey and Glass (1977).

$$\frac{dx}{dt} = \beta \frac{x_\tau}{1 + {x_\tau}^n} - \gamma x, \quad \gamma, \beta, n > 0, \tag{S1}$$

where x_τ represents the value of the variable x at time $(t - \tau)$. Depending on the values of the parameters, this equation displays a range of periodic and chaotic dynamics. For a commonly used setup where $\beta = 0.2, n = 10, \gamma = 0.1$, the system has a chaotic attractor for $\tau > 16.8$.

In the experiment setting, we pre-define a time series to graph mapping (e.g., Cosine similarity) and see if the model can predict the graph connectivity according to the historical time series. It makes sense because it has been proven that the vanilla ESN can predict the chaotic time series generated from nonlinear time-delay differential equations (e.g., Mackey-Glass).

In the experiment, we simulate a 5-node chaotic system. In total 10k chaotic time series data are simulated for each node as the "dynamics on graphs", with each time series having 500 timestamps.

1.2 Syn-Coupled

This synthetic dataset is for verifying if the proposed model can recover the graph structure from the nodes' time-series data under the assumption that the graph structure affects the interactions of time series. Random graphs are firstly generated with a pre-defined distribution. Each node of the graph generates base signals. The state of a node at a certain time point is determined by the node's base signal and the influence of its neighbors. When the graph is static, the signals on all the nodes will be synchronized. If we change the randomly generated graph structure every time l, the node signals will also be affected. Given the time series $S = S_1, S_2, ..., S_{n\times l}$ on all the nodes during $n \times l$, the experiment is to testify if the model can recover the graph structures $G = \{G_1, G_2, ..., G_n\}$

The dynamics of the weakly coupled time series which follow the governing equations:

$$\frac{d\theta_i}{dt} = \omega_i + \frac{K}{N} sin(\theta_j - \theta_i), \quad i = 1...N, \tag{S2}$$

where θ_i is is the signal on node i. Runge-Kutta method is used for generating the time series according to (S2)

We used the same external codeLaszuk (2017) base used by NRIKipf et al. (2018) for generating both Syn-Coupled-1 and Syn-Coupled-2. There are three initializing parameters for this implementation including

the initial phase $Y0$, intrinsic freq W, coupling matrix K. When s is a random sample from a uniform distribution over $[0, 1)$, the parameter settings are described as following.

- $Y0$: All the nodes are set to $s*2*\pi$ in Syn-Coupled-1. The first $Y0$ is set to $[0, \pi, 0, 1, 5]$ for each nodes in Syn-Coupled-2, then $Y0$ is set to the last phase in the last time series segment for the following time series segments.
- W: W is set to $s * 9 + 1$ in Syn-Coupled-1. W is set to $[28, 19, 11, 9, 2]$ (the default values in the repository) in Syn-Coupled-2.
- K: The edges in both datasets follow the Bernoulli distribution $p = 0.5$.

In the experiment, we simulate a 5-node interacting system of coupled oscillators. In total 10k chaotic time series data are simulated for each node as the "dynamics on graphs", with each time series having 100 timestamps.

1.3 Brain

As an example application to biological data, we use the resting-state fMRI data from over 1000 subjects released by the Human Connectome Project Smith et al. (2013). The subjects included in the dataset underwent an in total one hours high-resolution T1-weighted anatomical MRI scan. The smallest units measured in the raw data were $2 \times 2 \times 2$ mm voxels. The temporal resolution was around 0.7s. An anatomical parcellation is applied to the brain which yields 68 gyral-based ROIs, with 34 cortical ROIs in each hemisphere. The brain activities were measured with Blood-oxygen-level-dependent imaging in fMRI for each voxel but are averaged to the 68 ROIs. The averaged BOLD signals represent the 1-dimensional time series on each ROI. Functional connectivity is operationally defined as the temporal correlation between the time series of different brain regions.

1.4 Forum

As an example application to social media data, we use the healthcare forum data used by Gao et al. Gao et al. (2019). This dataset was collected from the Breast Cancer Community and covers an eight year period. The forum users' transition behavior patterns in different subforums are normally due to the changes in their health status and are reflected in their history activities. The users' transition behaviors can be modeled as sequences of transition graphs in which each node represents a subforum. In the experiment, we try to predict the users' accumulated transition graphs based on their history activity records.

1.5 Protein

As an example application to biology data, we use the protein folding data used in Anand and Huang (2018). Protein folding is the physical process by which a protein chain acquires its native 3-dimensional structure, a conformation that is usually biologically functional, in an expeditious and reproducible manner. During the protein folding process, the amino acids' position keeps changing. When the distance between two amino acid's distance is longer than a threshold, they can be viewed as unconnected. The connectivity of all the amino acids can form a dynamic graph. For graph learning, this can be considered as a graph of 8 nodes with node attributes (x, y, z) corresponding to 3D coordination of the atom of each amino acid, producing 300 temporal graphs with a sequence length of 100.

2 B. IMPLEMENTATION DETAILS AND EXPERIMENT SETTINGS

All experiments were conducted on a single 64-bit server with Nvidia TITAN V GPU.

2.1 Deep Echo-State Encoder

After the architecture of the ESN is optimized with NAS, the randomly generated ESN weights are normalized to meet a standard called Echo State Property (ESP) Jaeger (2001) as shown in Eq S3. This is a common practice being used in almost all the ESNs.

$$\begin{aligned} &\forall s = [u(1), u(2), ..., u(n)] \\ &\forall x, x' \in \mathbb{R}^{N_R} \text{ initial states} \\ &||\hat{\mathcal{R}}(s, x) - \hat{\mathcal{R}}(s, x')|| \to 0 \text{ as } n \to \infty \end{aligned} \tag{S3}$$

2.2 Dynamic Graph Topological Decoder

We tested message passing graph neural network (MPGNN) like in Kipf et al. (2018) as well as GAT as the dynamic graph topological decoder. In the experiment section, we only report the results with GAT but the results are very close with MPGNN.

The MPGNN module computes the graph adjacency/affinity matrix following the equation:

$$\begin{aligned} &v \to e : h_{i,j}^{(1)} = f_e^{(1)}([h_i^{(1)}, h_j^{(1)}]) \\ &e \to v : h_j^{(2)} = f_v^{(2)}(\sum_{i \neq j} h_{i,j}^{(2)}) \\ &v \to e : h_{i,j}^{(2)} = f_e^{(2)}([h_i^{(2)}, h_j^{(2)}]) \\ &... \\ &output : A_{i,j} = f_{out}(e_{i,j}^{(n)}) \end{aligned} \tag{S4}$$

here f are neural networks that map between the edge and node representations. We use fully-connected networks (MLPs) for the f functions.

Except for GAT and MPGNN, other options (e.g., MLP, RNN) can also be used for calculating pairwise node-to-node relationships in the last step and are compatible with our framework.

2.3 Deep-echo-state Architecture Optimization

As mentioned in Section 3.4, the search space is $A^{(R)} = [A^{(in)}, A]$, $A^{(R)} \in \mathbb{R}^{(d+R)\times R}$ where d is the input dimension and R is the size of the reservoir in ESN. In order to make the optimization possible with automatic differentiation, we relax the categorical edge choices to a continuous variable $A^{(R)}$ where $A_{i,j}^{(R)} := [0, 1]$. Similar approaches are used in NAS studies for image classification problems Liu et al. (2019); He et al. (2020). The NAS stage, we randomly select a node and use only the first 10% of time series data for NAS. The bi-level optimization of our NAS formulation is to optimize for making auto-regression with ESN. The intuition is that the encoder works well and can be used for other downstream tasks if it can make accurate auto-regression. After the continuous $A^{(R)}$ is optimized, we sample the discrete according to the lowest threshold λ to meet the ESP as mentioned in Section 2.1.

We tested different settings for the length of data used for NAS, ranging from 5% up to 100%. On most datasets, longer time series data don't help improve the encoder when the ratio reaches a threshold.

We chose 10% as a fixed ratio for all the datasets as it's a sweet spot for all the datasets considering the performance and search time.

REFERENCES

Anand, N. and Huang, P.-S. (2018). Generative modeling for protein structures. In *Proceedings of the 32nd International Conference on Neural Information Processing Systems*. 7505–7516

Gao, Y., Wu, L., Homayoun, H., and Zhao, L. (2019). Dyngraph2seq: Dynamic-graph-to-sequence interpretable learning for health stage prediction in online health forums. *2019 IEEE International Conference on Data Mining (ICDM)* , 1042–1047

He, C., Ye, H., Shen, L., and Zhang, T. (2020). Milenas: Efficient neural architecture search via mixed-level reformulation. In *Proceedings of the IEEE/CVF Conference on Computer Vision and Pattern Recognition*. 11993–12002

Jaeger, H. (2001). The "echo state" approach to analysing and training recurrent neural networks-with an erratum note. *Bonn, Germany: German National Research Center for Information Technology GMD Technical Report* 148, 13

Kipf, T., Fetaya, E., Wang, K.-C., Welling, M., and Zemel, R. (2018). Neural relational inference for interacting systems. In *International Conference on Machine Learning* (PMLR), 2688–2697

[Dataset] Laszuk, D. (2017). Python implementation of kuramoto systems

Liu, H., Simonyan, K., and Yang, Y. (2019). DARTS: Differentiable architecture search. In *International Conference on Learning Representations*

Mackey, M. C. and Glass, L. (1977). Oscillation and chaos in physiological control systems. *Science* 197, 287–289

Smith, S. M., Beckmann, C. F., Andersson, J., Auerbach, E. J., Bijsterbosch, J., Douaud, G., et al. (2013). Resting-state fmri in the human connectome project. *Neuroimage* 80, 144–168
